# *In vivo* osteoconductivity of surface modified Ti-29Nb-13Ta-4.6Zr alloy with low dissolution of toxic trace elements

**DOI:** 10.1371/journal.pone.0189967

**Published:** 2018-01-17

**Authors:** Eri Takematsu, Kimihiro Noguchi, Kensuke Kuroda, Toshiyuki Ikoma, Mitsuo Niinomi, Nobuhiro Matsushita

**Affiliations:** 1 Department of Biomedical Engineering, University of Texas at Austin, Austin, Texas, United States of America; 2 Department of Mathematics, Western Washington University, Bellingham, Washington, United States of America; 3 Institute of Materials and Systems for Sustainability, Nagoya University, Chikusa, Nagoya, Japan; 4 Department of Materials Science and Engineering, School of Materials and Chemical Technology, Tokyo Institute of Technology, Meguro, Tokyo, Japan; 5 Institute for Materials Research, Tohoku University, Aoba-ku, Sendai, Japan; 6 Department of Materials and Manufacturing Science, Graduate School of Engineering, Osaka University, Suita, Osaka, Japan; Universidad de Zaragoza, SPAIN

## Abstract

Simulated Body Fluid (SBF) has served as a useful standard to check the bioactivity of implant materials for years. However, it is not perfectly able to imitate human serum; sometimes disparities between the SBF test and animal test were confirmed. Therefore, to ensure the reliability of the results of the SBF test obtained from our previous study, an animal study was performed to check osteoconductivity of surface modified implant materials. Three types of solution processes, hydrothermal (H), electrochemical (E), and hydrothermal-electrochemical (HE), were performed on the Ti-29Nb-13Ta-4.6Zr alloy (TNTZ) to improve its bioactivity, and their bioactivities were measured *in vivo* using bone-implant contacts (BICs). BICs of the HE- and H-treated samples were significantly higher than that of the control. Metal ion diffusion towards the bone was also evaluated to examine the adverse effect of metal ions. No metal ion diffusion was observed, indicating the safety of our solution processed implant materials.

## Introduction

For the development of a new implant material, its bioactivity is a primary concern. To evaluate bioactivity in a simple and inexpensive way, the simulated body fluid (SBF) test was developed by Kokubo et al. [1]. This test assesses bioactivity of a sample through the formation of apatite on the sample. Since this is an easy and simple test, much research has relied on this test for checking bioactivity of samples. For example, NaOH-CaCl_2_-heat-water treated titanium alloy induced apatite formation in one day, implying its possible usage for implant materials [2]. However, several research groups have reported that the results of the SBF tests and animal tests contradict each other [2–5]. A recent review about the relationship between the SBF test and *in vivo* test highlighted that 8 out of 33 reports displayed different bioactivity results between the SBF test and *in vivo* test [6]. These disagreements are possibly due to differences between SBF and actual animal serum. In particular, SBF is different from human blood serum in terms of three important factors: i) the absence of proteins, ii) the presence of TRIS buffer in the SBF solution, and iii) the irregular carbonate content [7]. Human serum is composed of proteins that play important roles in osteointegration and carbonate, which acts as a pH buffer in serum. Thus, it is very important to consider these factors when checking the bioactivity of implant materials to minimize disparities with animal tests.

In our previous study, the SBF tests were conducted on a surface modified Ti-29Nb-13Ta-4.6-Zr (TNTZ) alloy [8]. This alloy is newly developed alloy for biomedical use due to its ideal Young’s modulus close to human bone and its high biocompatibility [9].

To improve its biocompatibility, three types of surface modification processes were performed in the previous study: hydrothermal (H), electrochemical (E), and hydrothermal-electrochemical (HE) processes [8]. One of the reasons for employing these solution processes is that they enable us to create metal oxides on the surface containing a lot of sodium ion, which facilitates the formation of apatite, leading to higher osteoconductivity. A second advantage is that they are environmentally friendly processes without using an energy consuming vacuum system. A third advantage is that it has good mechanical properties. In our previous study, these solution-processed TNTZ had a sufficient adhesive strength to be used as a biomaterial if we control the processing time properly [10]. In addition to those advantages, HE process has been known to create a fast-growing thin ceramic layer for several applications [11]. Therefore, it is also expected that fabrication time is much reduced than other processes such as the NaOH-CaCl_2_-heat-water treatment for the application of implant material.

Our previous report revealed that the order of apatite inductivity for the three processes was HE > H > E without impairing adhesive strength between alloy surface and apatite layer [8, 10]. However, these results were obtained without considering the *in vivo* environment, in which blood flow and proteins such as collagen exist. Therefore, in this paper, bioactivity of the TNTZ alloy surface modified by the three processes mentioned above was evaluated *in vivo* based on bone-implant contact (BIC). Moreover, we conducted a non-parametric statistical analysis on the BIC data of the TNTZ samples to provide a new insight into the treatment effects of these solution processes. It is noted that the statistical analysis presented in this paper is applicable to a wide range of biomedical studies where only small numbers of observations are available. Lastly, in addition to the BIC measurements, electron dispersive x-ray spectrometer (EDX) mapping was also performed to observe metal ion diffusion towards the bone region since metal ions were reported to cause bone resorption [12].

## Materials and method

### Material and sample pretreatment

The material used in this study was a forged TNTZ bar. The forged TNTZ bar was solutionized at 1073 K for 3.6 ks followed by water quenching. TNTZ samples with a thickness of 5 mm and a diameter of 2 mm were machined from the forged TNTZ bar subjected to solution treatment. The TNTZ samples were then degreased prior to the experiment by sonication in acetone and distilled water (Millipore Milli-Q, Merck Millipore; Germany) for 10 min each and air-dried at ambient temperature.

H, E, and HE processes were then performed on the TNTZ samples. All the samples were treated with 5 M NaOH solution containing 0.17 wt% of NH_4_F for 1 h. For the H and HE processes, the hydrothermal temperature was set at 90°C, and for the E and HE processes, the applied current density was set to 15 mA/cm^2^ [8]. All of the treated TNTZ samples were then stored in 5X PBS solution (1 to 2 dilution of 10X PBS, Thermo Fisher Scientific, Japan) at room temperature to retain the surface hydrophilicity until they were implanted [13].

### Surgical procedure

The TNTZ samples subjected to each solution treatment mentioned above and untreated control were implanted in the tibia metaphysis of seven-week old Sprague Dawley rats (Charles River Japan, Yokohama) weighing 330–360 g. Bioactivities were evaluated from new bone formation. Before surgery, all the TNTZ samples were cleaned with distilled water. Rats were kept in quarantine for seven days. Prior to surgery, the rats were anesthetized with pentobarbital (25–30 mg/kg), and the operation region was shaved and cleaned with povidone-iodine solution and ethanol. Holes with a diameter of 0.5 mm and a length of 2 mm were created at the middle of the tibia metaphysis using a slow speed rotary drill, and then the TNTZ samples were inserted in the hole. To prevent festering of the operation site, Penicillin G was administered. Then, the skin was sutured and cleaned with povidone-iodine solution.

After surgery, the rats were kept individually in polycarbonate cages and reared for two weeks. No complications or ill effects were observed while rearing. Rats were then sacrificed by exsanguination being anesthetized with 2–3% isoflurane and the implanted TNTZ samples with surrounding tissues were explanted. The TNTZ samples were fixed in 10% neutral buffered formalin solution followed by rinsing with distilled water. Then, the rinsed TNTZ samples were dehydrated in a graded series of ethanol, followed by acetone, and embedded in methylmethacrylate. Subsequently to polymerization, each TNTZ sample was mechanically sliced to be 20 μm thick using a microcutting machine (BS-3000N, EXAKT GmbH, Germany) and a microgrinding machine (MG-4000N, EXAKT GmbH, Germany). The sliced sample was then stained with toluidine blue and observed by light microscopy to determine BIC. This experiment was carried out at the laboratory of Hamri Co., Ltd. (Ibaraki, Japan), which has been approved by the Association for the Assessment and Accreditation of Laboratory Animal Care (AAALAC) International. The experimental protocol was approved by the Institutional Animal Care and Use Committee (IACUC) of Hamri Co., Ltd. (Ibaraki, Japan).

### Measurement of BIC

BIC was determined by the linear measurement of direct bone contact with the implant surface, which was calculated using the following formula:

$$BIC=\frac{The\;sum\;of\;the\;length\;of\;the\;bone\;formation\;on\;the\;implant\;surface}{Total\;inserted\;implant\;length}\times 100$$ (1)

BIC was measured separately in cancellous and cortical bone regions using a fluorescence microscope (Olympus BX-51-33PH-SP, Japan) (n = 3-6/group). Statistical analysis was then performed to determine the treatment effects of different solution processes.

### Statistical analysis

Statistical analysis was performed on the BIC data of the TNTZ samples with and without each alkali solution treatment in the cancellous bone region and is detailed below in the following subsections.

#### Outlier detection

Due to small sample sizes, outlier detection was performed by R using Hampel’s identifier [14]; the statistic is commonly referred to as the modified Z-score [15]. Hampel’s identifier utilizes robust measures of center (median) and scale (median absolute deviation), and hence it is a more reliable metric compared to the raw Z-score or methods based on sample mean and sample standard deviation, especially for small sample sizes. Any observation exceeding the cut-off value of 3.5, as recommended by Iglewicz and Hoaglin (1993) [15], was declared as an outlier in our study.

#### Pairwise comparisons of treatments

With such small sample sizes, it was not possible to assume any underlying distribution of the data. Therefore, a robust non-parametric rank-based multiple contrast testing procedure [16] was performed using the nparcomp package in R [17]. The procedure above assessed the overall significance and pairwise comparison results at the same time without any contradiction based on the concept of simultaneous test procedure [18]. It is robust to non-normal distributions, small or unequal sample sizes, and unequal variances while maintaining a high power [16]. It is important to note that non-parametric procedures utilize relative effects rather than means to compare samples.

#### Effect sizes

Effect sizes, which are used to supplement the results from statistical tests, are reported to provide a more intuitive understanding of the magnitude of differences between samples. To facilitate the understanding of its magnitude, we employed the Hasselblad-Hedges effect size, which is useful for relative effects [19]. Specifically, the formula is:

$$C(p_{1},p_{2})=\frac{1}{1.81}ln(\frac{p_{1}/(1-p_{1})}{p_{2}/(1-p_{2})})$$ (2)

where *p*_1_ and *p*_2_ are the relative effects of the two samples being compared. the Hasselblad-Hedges effect size allowed us to interpret the magnitude of effects easily due to its similarity to one of the most widely used effect sizes called Cohen’s *d*. Specifically, the common cut-off values of 0.2, 0.5, and 0.8 in absolute values were respectively used as small, medium, and large effects in this study. Statistical significance was determined at *p* < 0.05, where *p* denotes the adjusted *p*-value.

### Analysis of metal ion dissolution

The interface region of bone and TNTZ on the sliced implanted sample was observed using a scanning electron microscopy (SEM) (Hitachi S 450, Japan) with an acceleration voltage of 15 kV. To confirm any metal ion diffusion from the TNTZ alloy into surrounding tissue, the interface region of the bone and TNTZ alloy on the sliced implant was analyzed using an energy-dispersive X-ray spectrometer (EDX, JEOL JSM-5410LV, Japan). Then, elemental mappings of Ca, Ti, Nb, Ta, and Zr were conducted. For surface chemistry analysis, X-ray photoelectron spectroscopy (XPS) was performed in a PerkinElmer 5500MT spectrometer. XPS data were acquired using Al Kα X rays with pass energy 8 kV. Sputter cleaning was done with Ar^+^ sputter gun. MaltiPack software was used for the analysis.

## Results and discussion

### Results

#### BIC

**Fig 1:**
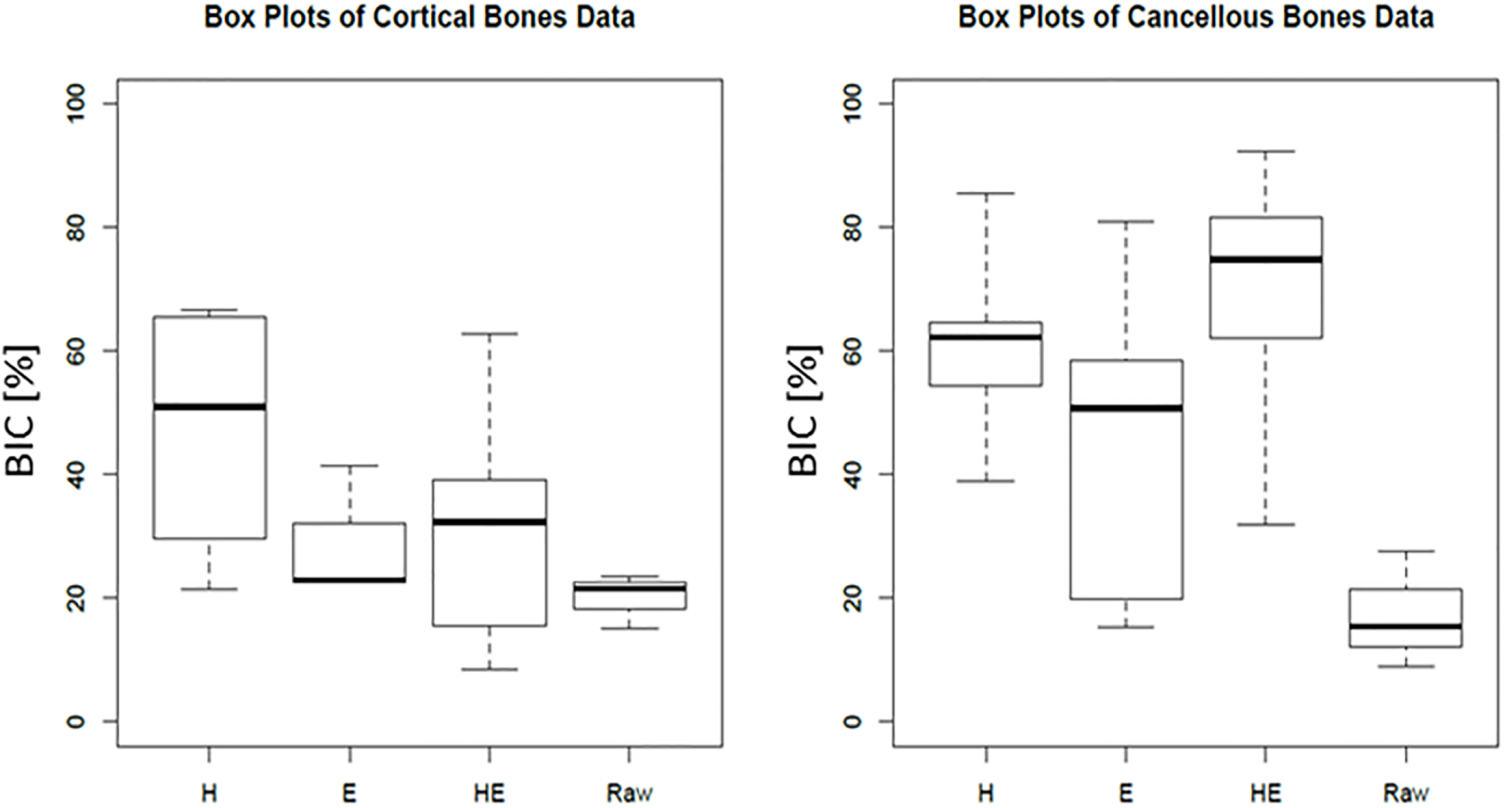
Box plots of BICs of TNTZ samples without (raw), and with H, E, and HE processes (H, E, and HE, respectively) in the (a) cortical and (b) cancellous bone region.

No significant differences were observed in the cortical bone region, while significant differences were detected in the cancellous bone region between samples-Box plots of BICs of the TNTZ samples without the alkali solution treatment (referred to as raw) and with each alkali solution treatment in the cortical bone and cancellous bone region are shown in Fig 1. The bottom and top of the box show the first and third quartiles (bottom 25% and top 25%), respectively, and the middle bold line in the box shows the median. The top and bottom horizontal lines of the vertical dotted lines for each box exhibit the minimum and maximum values of each sample, respectively.

In the cortical bone region, there were only minor differences in the BIC among the TNTZ samples without and with alkali solution treatments, and the median of each TNTZ sample was between 20–50%. On the other hand, BIC in the cancellous bone region showed clear differences among the TNTZ samples with and without alkali solution treatments. The TNTZ samples subjected to the HE, H, and E processes had much higher median values of 78, 62, and 51%, respectively, than the raw TNTZ sample (15%). Such a different tendency in the BIC values between the cortical and cancellous bone regions may arise from blood flow differences in these two regions. It is widely known that the blood flow is much faster in the cancellous bone than in the cortical bone [20]. Therefore, healing of the cancellous bone is faster than that of the cortical bone. Moreover, the implanted period of the TNTZ samples in this study was two weeks. Thus, it is difficult to observe the differences in BICs of the TNTZ samples in the cortical bone region where the healing was relatively slow. For the reasons mentioned above, analyzing BIC in the cancellous bone was appropriate for evaluating the difference in the osteoconductivity among the TNTZ samples with and without each alkali solution treatment.

#### Statistical comparisons

Pairwise and effect size comparisons clearly show the difference in each treatment- No outlier was detected using Hampel’s identifier. As such, all of the observations were used in the analysis. Specifically, there were 5, 5, 6, and 3 observations from the TNTZ samples subjected to H, E, and HE processes, and the raw TNTZ sample for the cancellous bones, respectively.

Table 1 summarizes the adjusted *p*-values for the all-pairwise differences in the BICs of the cancellous bones. The *p*-values were adjusted appropriately for multiplicity using the multivariate *t*-distribution, and statistical significance was determined at *p* < 0.05. In particular, statistically significant evidence of alkali solution treatment effects was found when the TNTZ sample subjected to the H process (H) and the raw TNTZ sample (Raw) were compared, and also when the TNTZ sample subjected to the HE process (HE) and the raw TNTZ sample (Raw) were compared.

**Table 1:** Summary of adjusted *p*-values for the all-pairwise comparisons of the cancellous bones for the TNTZ samples subjected to H, E, and HE processes and the raw TNTZ sample (Raw).

Cancellous *p*-value	H	E	HE	Raw
H	-	0.578	0.884	0.007
E	-	-	0.319	0.311
HE	-	-	-	0.005
Raw	-	-	-	-

A question that arose from these findings was if there was any practical difference between the TNTZ samples subjected to H and E, HE and E, and H and HE. Even though the pairwise comparisons above revealed no statistically significant difference, this may be due to the low power of detecting any statistically significant difference for very small sample sizes. As a remedial measure, the Hasselblad-Hedges effect size was also calculated for measuring the practical magnitude of pairwise differences. Table 2 summarizes the Hasselblad-Hedges effect size for the cancellous bones. In the table, both the effect sizes measured by *C*(*p*_1_, *p*_2_) and the corresponding magnitude of effect for the six all-pairwise comparisons were reported. Several medium and large effects were observed by using the effect size *C*(*p*_1_, *p*_2_). For the cancellous bones, the difference between the TNTZ samples subjected to H and Raw, E and HE, E and Raw, and HE and Raw, were considered either medium or large.

**Table 2:** Summary of *C*(*p*_1_, *p*_2_) for the all-pairwise comparisons of the cancellous bones or the TNTZ samples subjected to H, E, and HE processes (H, E, and HE respectively) and the raw TNTZ sample (Raw).

Cancellous *C*(*p*_1_, *p*_2_)	H	E	HE	Raw
H	-	0.456 (Small)	-0.225 (Small)	1.191 (Large)
E	-	-	-0.681 (Medium)	0.735 (Medium)
HE	-	-	-	1.416 (Large)
Raw	-	-	-	-

The analysis above suggested advantages of some methods, which were not clear from the statistical tests. In particular, the effect size of -0.681 for the pair E and HE showed a medium advantage of HE over E. Moreover, the comparison between H and HE resulted in the effect size of -0.224, indicating a small advantage of HE over H. From these observations, some advantage of the HE treatment over H and E was suggested. In addition, the ones that showed statistically significant differences (H vs. Raw and HE vs. Raw) had large effects (1.191 and 1.416, respectively), implying substantial advantages of the H and HE treatments over the untreated TNTZ sample.

#### Diffusion of alloying elements

**Fig 2:**
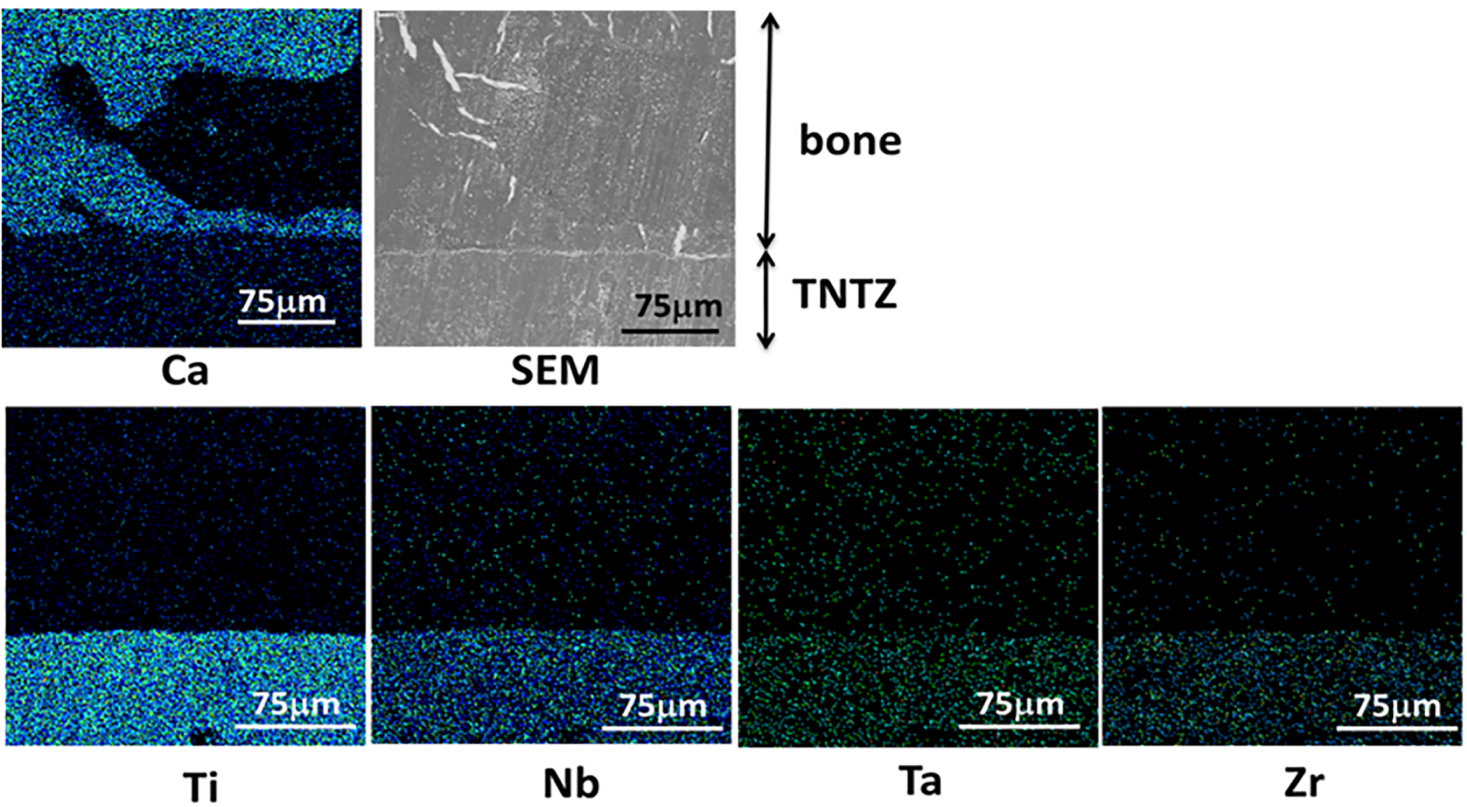
EDX mappings of Ca, Ti, Nb, Ta and Zr at interface between the TNTZ sample subjected to H and bone along with SEM image.

**Fig 3:**
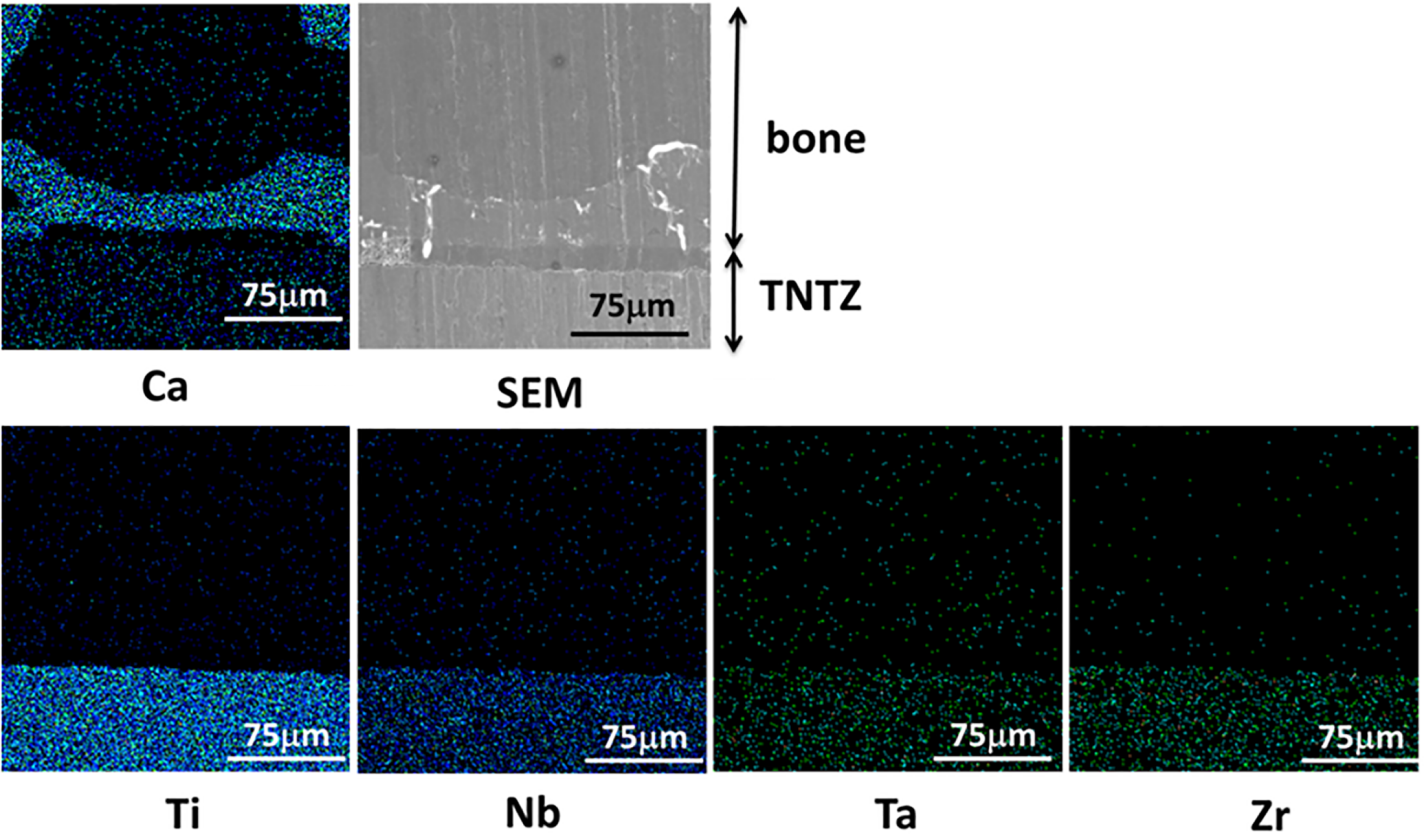
EDX mappings of Ca, Ti, Nb, Ta and Zr at interface between the TNTZ sample subjected to E and bone along with SEM image.

**Fig 4:**
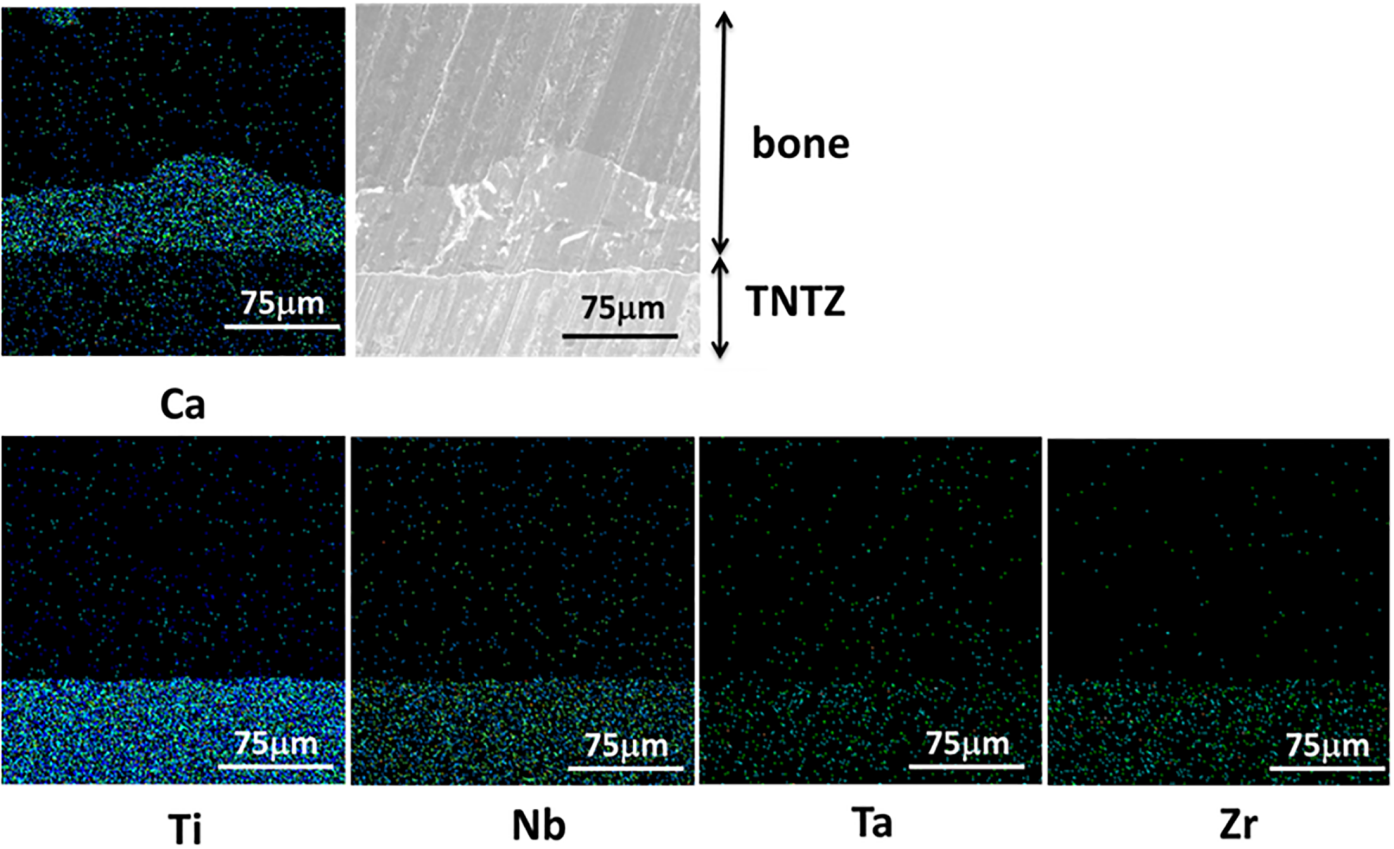
EDX mappings of Ca, Ti, Nb, Ta and Zr at interface between the TNTZ sample subjected to HE and bone along with SEM image.

No alloying elements diffused into bone- Figs 2–4 show EDX mapping on the surface of the TNTZ sample subjected to each alkali solution treatment. Ca indicates the bone region, and Ti, Nb, Ta, and Zr indicate the alloying elements. Metal ions were under the detection limit in the bone region, implying that there was less metal ion diffusion towards the bone region. The bone directly connected with the TNTZ samples subjected to H and HE as shown in Figs 2 and 4. However in Fig 3 (E), only a small part of the bone connected with the TNTZ sample subjected to E. Therefore, EDX mapping revealed poor osteoconductivity of the TNTZ sample subjected to E.

### Discussion

#### BIC

**Fig 5:**
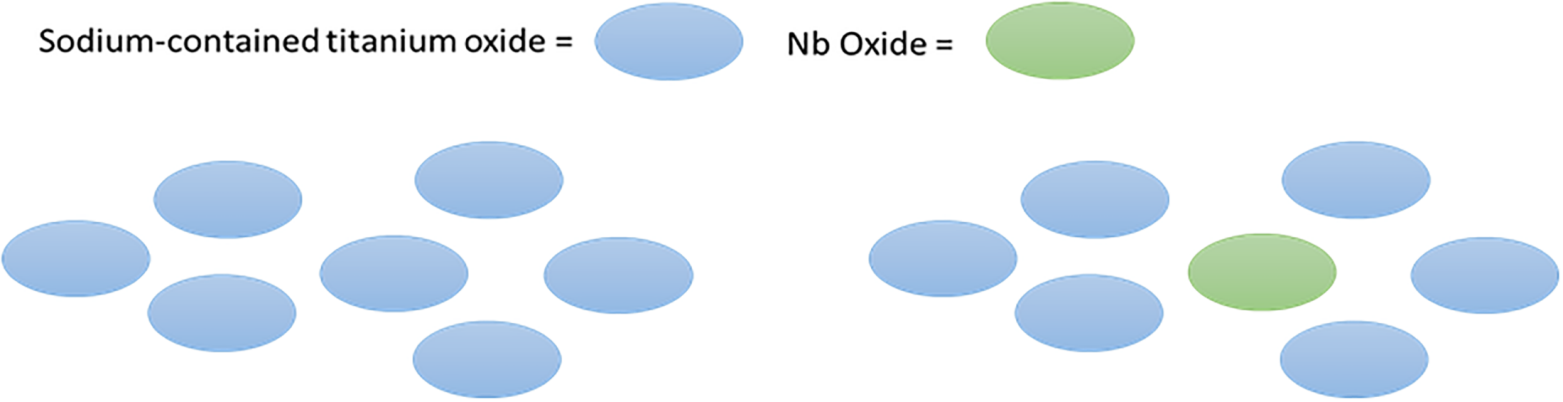
Schematic illustration of the position of oxides. Left: when treated by the H and HE method. Right: when treated by the E method.

In the cancellous bone region, statistical analysis of BIC indicated that the TNTZ samples subjected to the H and HE processes had significantly higher BIC values than that of the raw TNTZ sample (*p* < 0.01) while the BIC value of the TNTZ sample subjected to the E process was not significantly different from that of the raw TNTZ sample. These differences could be attributed to the chemical composition of the sample surface. Our previous experimental results suggested that the sample surface with higher Nb oxide is likely to inhibit apatite formation, and a higher amount of Nb was found on the surface of the TNTZ subjected to the E process [8]. In this study, it was also confirmed by XPS that a higher amount of Nb was found on the surface of the TNTZ sample subjected to the E process; a lower amount of Nb was found on the surfaces of the TNTZ samples subjected to the H and HE processes. Table 3 shows the Nb/Ti ratio of the TNTZ sample subjected to the E process is more than twice as much as those of the TNTZ samples subjected to the H and HE processes. Our previous finding [8] has confirmed that Nb on the surface of the TNTZ sample exists as Nb oxide. The reason why Nb oxide is likely to exist on the surface of the TNTZ sample has not been determined yet. A previous report [8] revealed that surface oxides were mainly Na-contained amorphous titanium oxides, where Ti^4+^ ion was coordinated with oxygen and stable with some Na^+^ ions. When the TNTZ samples were subjected to the H and HE processes, their surfaces were mainly composed of the Na-contained amorphous titanium oxide. However, for the TNTZ sample subjected to the E process, Nb oxides took up spaces where Na-containing amorphous titanium oxides existed, as shown in Fig 5. Here, the Nb oxide does not contain Na^+^. As a result, the total amount of Na^+^ ions decreases. Therefore, the TNTZ sample subjected to the E process contained less Na^+^ ions on its surface, which releases less Na^+^ ions in blood plasma, resulting in a less negatively charged surface as compared to those of the TNTZ samples subjected to the H and HE processes. As a result, the surfaces of the TNTZ samples subjected to the H and HE processes were very much negatively charged and could attract Ca^2+^ ions easily. Once Ca^2+^ ion is adsorbed on the surface, fibronectin, which is considered as an important cell adhesion molecule, binds with the surface via a Ca^2+^ site by its carboxyl group [21]. Fibronectin adsorbs more on the surfaces of the TNTZ samples subjected to the H and HE processes, leading to higher osteoblast cell adsorption and a higher BIC value. The tendency of the BIC values matched with that of the *in vitro* experiment, which analyzed osteoconductivity using an SBF immersion test [8]. According to this study, apatite inductivity was the highest for the TNTZ sample subjected to the HE process, followed by the H and E processes. This tendency corresponded with the results from the *in vivo* experiment and supported the usefulness of the immersion test in SBF solution as an indicator of osteoconductivity.

**Table 3:** Surface chemical compositions (in mass %) and ratio of Nb content to Ti content (Nb/Ti) of formed layers on the raw TNTZ sample (Raw) and the TNTZ samples subjected to the H, E, and HE processes (H, E, and HE, respectively).

	Na	O	Ti	Nb	Ta	Zr	Nb/Ti
Raw	0.00	68.99	21.31	7.59	0.58	1.52	0.36
H	28.54	52.55	16.78	1.67	0.35	0.10	0.10
E	19.67	57.69	17.50	4.30	0.61	0.22	0.25
HE	25.81	54.30	17.58	1.76	0.33	0.23	0.10

Unit is %.Nb/Ti is a ratio.

#### Alloying elements

Several studies showed adverse effects of the Ti^4+^ ions release on bone remodeling. Mine et al. suggested that the Ti ion inhibited the differentiation of osteoblasts and altered the ratio of RANK ligand and osteoprotegerin (OPG) gene expression, which caused bone resorption at the interface of dental implant and tissues [22]. Other reports showed that only a small amount of Ti altered the behavior of macrophage-like RAW264 cells to enhance phagocytosis, which caused oxidative stress and inflammation [23]. Therefore, it is imperative to investigate any metal ions diffusion towards the bone region. EDX mapping results showed metal ion diffusions towards bone region that is less than a detection limit, which indicates that there is no or little amount of metal ion diffused to bone. This suggests the possible usage of alkali treated TNTZ as a safe implant material. The reason why the TNTZ samples subjected to alkali solution treatment showed good corrosion resistance was speculated because the fabricated oxide layer was a thicker amorphous layer. The previous report by Mizutani revealed that a thick oxide layer prevented the dissolution of any metal ion [24]. The thicknesses of the surface layers fabricated on the TNTZ samples subjected to the E, H, and HE processes were 0.65, 0.78, and 2.26 μm, respectively. Such thick oxide layers may prevent metal ion diffusion as earlier stated. Moreover, it can be said that amorphous passive layers have a good corrosion resistance because they hardly contain grain boundaries or structural defects [25, 26]. According to the previous report [8], fabricated oxide layers are composed of an amorphous phase, which may serve as a good passive layer. From these factors, little of no metal ion diffusion was observed on the TNTZ sample subjected to alkali solution treatment. However, this is a bulk level study, therefore further study will be needed to scrutinize the cytotoxic effect of diffusion elements from TNTZ.

## Conclusion

In summary, the bioactivity of TNTZ, whose surface was modified by alkali solution treatments (H, E, and HE processes), was evaluated *in vivo* based on the bone-implant contact (BIC) test and deemed as safe biomaterials. The HE process, in particular, is a very promising surface modification process for TNTZ as it results in high osteoconductivity and corrosion resistance. Specifically, the degree of osteoconductivity *in vivo* matched with the results from the *in vitro* experiment [8], supporting the usefulness of the immersion test in SBF solution as an indicator of osteoconductivity in this study. Also, our statistical analysis of the BIC data using recent non-parametric hypothesis tests and the Hasselblad-Hedges effect size empirically support the conclusion. It is noted that the statistical analysis presented in this paper could be highly applicable to other studies in biomedical research where small sample sizes are prevalent. The present results suggest that HE treated TNTZ alloy may be helpful for developing novel orthopedic implant due to its great osteoconductivity and corrosion resistance.
